# A Review on Nanomaterials for Improving the Electrochemical Performance of the Next-Generation Lithium-Ion Batteries

**DOI:** 10.3390/nano16181174

**Published:** 2026-09-17

**Authors:** Vikas Kumar, Shiv Lal, Pushpendra Kumar Singh Rathore, Rohit Kumar Singh Gautam

**Affiliations:** 1 Department of Mechanical Engineering, University Department, Rajasthan Technical University, Kota 324010, India; 2 Department of Mechanical Engineering, Manipal University Jaipur, Jaipur 303007, India; 3 Integrated Centre for Energy and Sustainable Technologies, Manipal University Jaipur, Jaipur 303007, India; 4 Department of Mechanical Engineering, Teerthanker Mahaveer University, Moradabad 244001, India

**Keywords:** nanomaterials, Lithium-Ion Batteries, specific capacity, solid state electrolyte, ionic conductivity, coulombic efficiency

## Abstract

The rapid development of electric vehicles, portable electronics, and renewable energy storage has stimulated the demand for Lithium-Ion Batteries (LIBs) with higher energy density, better rate capabilities, enhanced cycling stability, improved safety, and fast-charging capabilities. Nanomaterials have emerged as an effective approach to address the limitations of traditional LIB components owing to their high surface area, shortened ion diffusion pathways, adjustable structures, and improved electrochemical performance. This review critically evaluates recent developments in nanomaterials for LIBs, focusing on their applications in anodes, cathodes, and electrolytes. Anodes including silicon, carbon-based nanomaterials, transition-metal oxides, binary metal oxides (BMOs), and binary transition-metal oxides (BTMOs) are discussed in terms of capacity, reaction kinetics, and structural stability. Nanostructured cathodes, including lithium iron phosphate, nickel–manganese–cobalt oxides, transition-metal sulfides, and zinc–manganese oxides, are evaluated with respect to lithium-ion transport, rate capability, capacity retention, and structural stability. Nanomaterial-modified liquid and solid-state electrolytes are further examined. Moreover, this review discusses also the challenges that accompany the use of nanomaterials. Consequently, future research should prioritize the need to overcome the challenges to translating nanoscale materials advances into safe, durable, high-performance, and commercially viable next-generation LIBs.

## 1. Introduction

The rechargeable Lithium-Ion Batteries (LIBs) are utilized in electric vehicles and portable electronic items. It stores and releases energy through ion transport between an anode and a cathode while charging and discharging. Generally, the traditional electrode materials face some challenges, such as low specific capacity, volume expansion, and formation of dendrites; limited cycle life; charging/discharging rates; energy density; and stability. The nanomaterials have emerged as an effective approach for overcoming these challenges, primarily because of their exceptional physicochemical properties associated with nanoscale dimensions.

The high specific surface area of nanomaterial triggers the electrochemical reactions and, hence, improves capacity and provides faster kinetics. The enhanced conductivity of nanomaterials allows for quicker ion movement, reducing charging durations and thereby allowing for faster charging times [1]. Nanomaterials exhibit better cyclability and minimize volume changes while charging and discharging, leading to a longer lifespan for the battery [2]. Additionally, the nanomaterials improve the charging and discharging rate due to the shortened diffusion paths for lithium ions and electrons. However, the nanomaterials significantly enhance energy density, leading to higher energy storage capabilities, which are necessary for applications in EVs and portable electronics [3].

Z. Cui [4] studied various nanomaterials for increasing the electrochemical performance of LIBs and found that electrochemical performance is significantly enhanced. They have also observed an improved specific capacity, stability, and cycling ability. To enhance electronic and ionic conductivity, as well as capacity retention and rate capability in LIBs, Choi et al. [5] investigated the addition of multi-walled carbon nanotubes (MWCNTs) to high-nickel cathode materials. According to their results, MWCNT lowers an internal resistance in cathodes and increases the ionic conductivity. It was found that the capacity retention over cycle life was 89.5%. L. Lyu [6] investigates the use of graphene, carbon nanotubes, and silicon nanomaterials to improve the performance of LIBs, focusing on enhancements in capacity, charge rate, and cycle life due to their outstanding physical and chemical properties. Incorporating nanomaterials into the electrodes of LIBs significantly enhances their performance. This enhancement is attributed to the electrodes’ higher surface area, improved ionic conductivity, and overall increased battery capacity [7,8]. However, the nanostructured electrodes enable faster ion diffusion and boast large specific capacities, allowing for shorter charging times and improved longevity. Silicon-based nanostructures can significantly improve the theoretical capacity of anodes for LIBs, which might overcome the limitations of the energy density as compared to traditional graphite anodes [9]. The zinc oxide–polyaniline (ZnO-PANI) nanocomposite demonstrates enhanced battery performance through its high specific capacity of 1052 mAhg^−1^, excellent Coulombic efficiency (~98%), and impressive capacity retention of 92.99% after 500 cycles, making it suitable for LIB applications [10]. However, there are some methods, including nano-structuring, nano-doping, and surface coating, that improve stability, capacity rate, and cycle performance for cathodes and anodes, thus facilitating faster charge/discharge rates and structural stability [11]. Additionally, the next-generation batteries are crucial in today’s market because they strive to offer a blend of higher energy density, faster charging, longevity, enhanced safety, affordability, and sustainability. This makes them suitable for electric vehicles, electronics, and various emerging energy technologies. The integration of nanomaterials further elevates the relevance of these advanced batteries by improving energy density, capacity rates, cycle life, safety, and material efficiency. This advancement plays a key role in bridging the gap between laboratory achievements and real-world battery applications.

The objective of this review is to systematically study the role of nanomaterials in enhancing the electrochemical performance of Lithium-Ion Batteries(LIBs) by critically examining their applications in anodes, cathodes, liquid electrolytes, and solid-state electrolytes. Particular emphasis is placed on correlating nanomaterial characteristics, structural properties, and interfacial interactions with key battery-performance parameters, including specific capacity, rate capability, ionic conductivity, Coulombic efficiency, cycling stability, and safety. This review further assesses the mechanisms by which nanomaterials improve Li^+^ transport, electrode–electrolyte interactions, structural stability, and dendrite suppression, while comparing liquid and solid-state electrolyte systems. Finally, this review identifies the remaining scientific and technological challenges, including interfacial instability, agglomeration, long-term durability, cost, scalability, and environmental sustainability, and proposes future research directions toward safe, high-performance, scalable, and commercially viable next-generation LIBs.

## 2. Fundamental of Lithium-Ion Battery (LIB)

**Figure 1:**
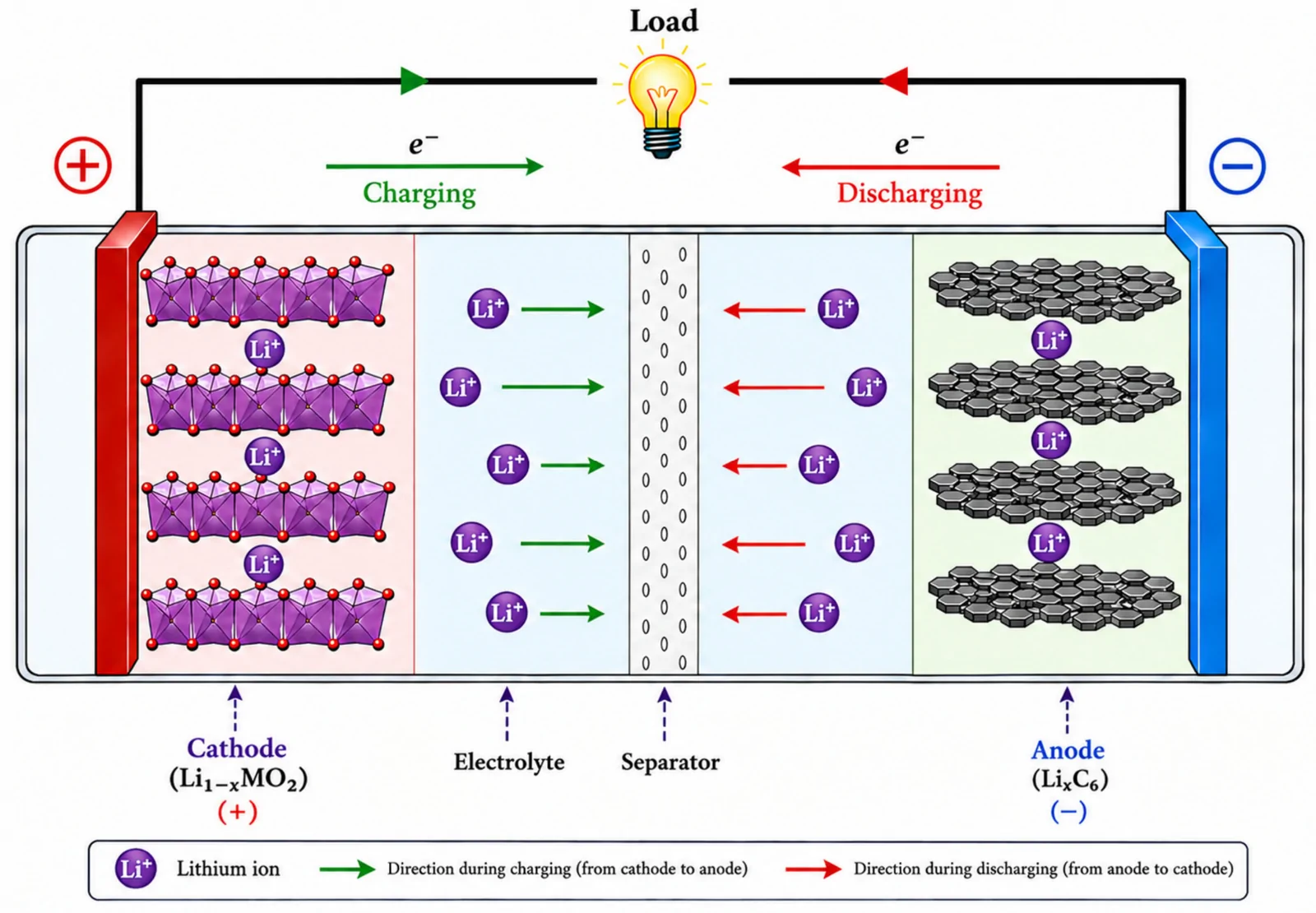
A typical schematic of a LIB. Data from Ref. [15].

LIBs are rechargeable sources of power due to reversible flow of ions. During the discharge process, ions travel from the anode to the cathode through the electrolyte, and during charging, they move in the opposite direction [12]. High energy density is the key feature of LIBs, creating more energy storage within a small volume compared to other batteries. This feature makes them suitable for consumer appliances like laptops, tablets, and smartphones. Moreover, LIBs are in demand for electric vehicles due to their being lightweight and having a high power output. LIBs are playing a major role in the global movement toward eco-friendly transportation. LIBs are cost-effective and reliable due to their high efficiency (~95%) and better life cycle over time. Fast charging and discharging make them ideal for balancing non-conventional sources of energy also. A typical schematic of a LIB is presented in Figure 1. It consists of following elements:

- Cathode: A positive electrode made of LCO, LIP, or NMCs.
- Anode: A negative electrode made of graphite.
- Electrolyte: Medium of ionic conductivity (lithium organic solvent).
- Separator: A porous media between cathode and anode to avoid short circuit.

In LIBs, the following reactions, as described by Equations (1)–(3) [13,14], take place during charging.

Anode reaction:

(1) $$C_{6}+Li^{+}+e^{-}\overset{Ch\arg ing}{\to }LiC_{6}$$

Cathode reaction:

(2) $$LiCoO_{2}\overset{Ch\arg ing}{\to }Li^{+}+e^{-}+CoO_{2}$$

Final reaction:

(3) $$LiCoO_{2}+C_{6}\overset{Ch\arg ing}{\to }LiC_{6}+CoO_{2}$$

The above reaction reversibly takes pace while discharging.

## 3. Mechanism of Nanomaterials

Nanomaterials are substances that have structures, characteristics, or properties at the nanometer level (usually in the range of 1~100 nm). These materials stand out from others due to their exceptional attributes. Nanomaterials significantly improve energy storage technologies, particularly improving battery efficiency. Unlike bulk materials, nanomaterials possess unique physical, chemical, and electrochemical characteristics at the nanoscale (generally between 1 and 100 nm). A comprehensive explanation of the mechanisms of nanomaterials that contribute to their importance is outlined as follows.

### 3.1. Increased Surface Area

Nanomaterials have a larger specific area and more active sites for electrochemical reactions. It helps in faster electron transfer and lithium-ion diffusion. As a result, the battery can charge and discharge more rapidly.

### 3.2. Improved Electrical Conductivity

Nanomaterials like graphene and carbon nanotubes demonstrate outstanding electrical conductivity. As a result, internal resistance is minimized. This also enhances the energy density and, hence, performance of electric vehicles.

### 3.3. Shorten Diffusion Paths

In nanostructured materials, ions and electrons move over shorter distances. This enhances the intercalation and de-intercalation processes of ions. These processes are critical for energy storage systems like lithium-ion batteries. Additionally, the batteries can be charged and discharged rapidly without any noticeable drop in performance.

### 3.4. Mechanical Stability and Flexibility

The nanomaterials can better accommodate the changes in volume during charging or discharging. This results in a longer life cycle and structural durability.

### 3.5. Enhanced Electrochemical Stability

**Figure 2:**
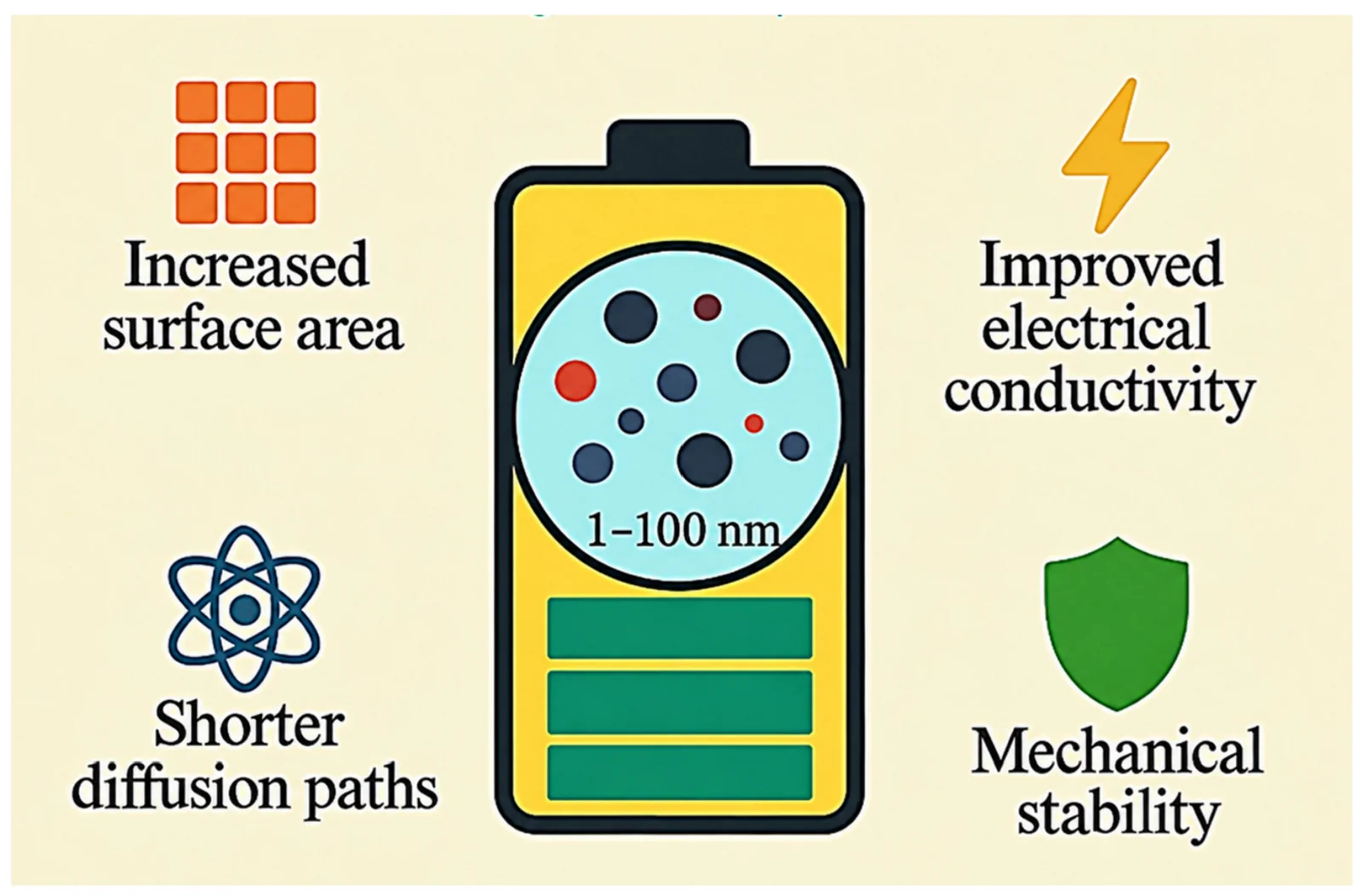
Mechanism of nanomaterials in LIBs.

Core–shell nanostructures can be utilized to create surface coatings that stabilize reactive materials. This improves safety by minimizing the risk of thermal runaway or short circuits. Additionally, it prevents any unwanted interactions between the electrolyte and the electrode. In energy storage applications, nanomaterials are utilized in different parts of batteries, such as the anode, cathode, electrolyte, and separators. Generally, integrating nanomaterials into batteries results in increased energy and power density, quicker charging times, extended lifespan, enhanced safety, and improved mechanical and chemical stability. Figure 2 depicts how nanomaterials enhance the performance of batteries.

A key factor contributing to the enhanced electrochemical performance of nanomaterials in LIBs is the shortened diffusion path, as outlined in the previous discussion. However, there are some disadvantages also involved with LIBs while using nanomaterials, which are as follows.

### 3.6. Increased Parasitic Reactions

Parasitic reactions in LIBs are undesirable electrochemical processes that waste active lithium ions, damage the electrolyte, and lead to irreversible capacity loss and self-discharge. These reactions reduce Coulombic efficiency and life of the battery.

### 3.7. Solid Electrolyte Interphase Formation

The Solid Electrolyte Interphase (SEI), created when the electrode surface in LIBs undergoes passivation. It is crucial for determining both the life and performance of the battery. SEI behave like an electronic insulator and reduces the decomposition of electrolyte [16]. It also increases the resistance and impedance of the battery. The SEI layers are formed on the anode of the LIBs.

### 3.8. Reduced Initial Coulombic Efficiency

The active lithium ions (Li^+^) are consumed permanently while first charging LIBs. This reduces the initial Coulombic efficiency (ICE). The Li^+^ does not return to the cathode during the discharging. The main causes of the reduced ICE are trapping of Li^+^, formation of SEI, surface side reactions, and irreversibility of electrode structures.

### 3.9. Particle Agglomeration

Nanomaterials have a tendency to agglomerate. It is the major challenge for electrochemical energy systems. The agglomeration of nanomaterials deteriorates the transport and electrochemical properties by reducing the effective surface area. The main causes of nanomaterials’ agglomeration are strong Van der walls forces, Brownian motion, high concentration of nanomaterials, poor dispersion, ionic strength, and long-term stability. Due to the factors, the agglomeration diffusion pathway of Li^+^ increases and thus reduces the rate capability and cycle stability.

### 3.10. Processing Complexity

**Figure 3:**
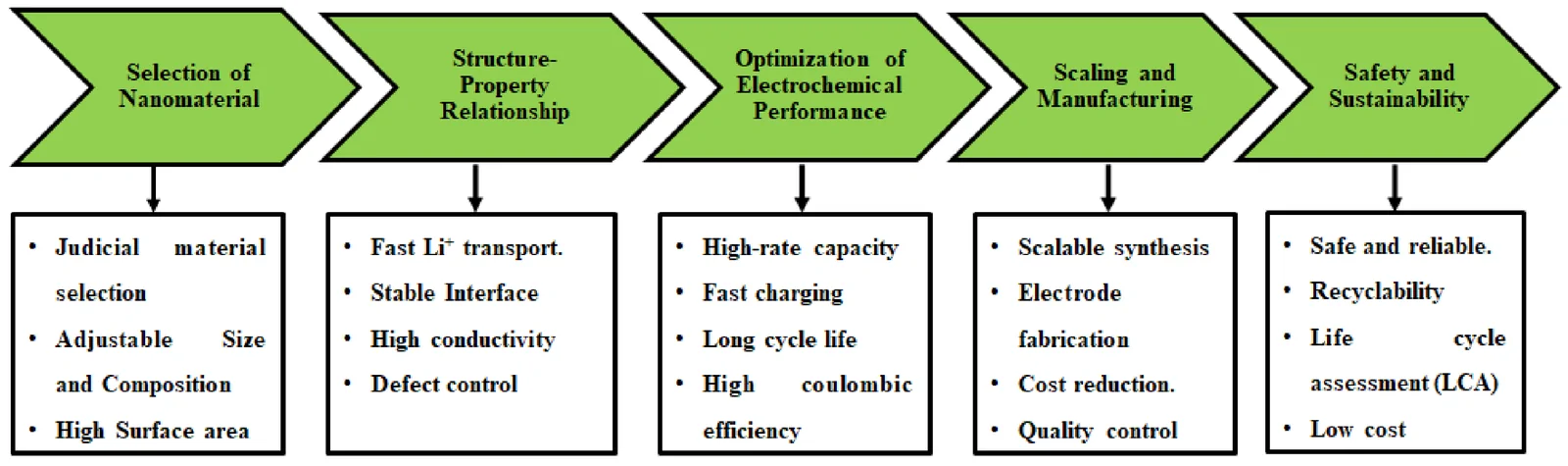
A systematic approach toward performance and sustainability of LIBs.

The addition of nanomaterials into the LIB’s electrode can improve the electrochemical performance, but, at the same time, the processing of nanomaterials is very complex compared with micro-size particles. The major challenges in the processing of nanomaterials are dispersion, controlling of agglomeration, synthesis of particle size, electrode processing, scaling, and costing of characterization. However, a tentative roadmap for nanomaterials toward high-performance and sustainable LIBs is presented in Figure 3. Moreover, quantitative benchmarks typically for next-generation LIBs could be as per given in Table 1.

## 4. Nanomaterials in Lithium-Ion Batteries

Lithium-Ion batteries (LIBs) have certain disadvantages, including poor energy density, the least cycle life, and safety problems. Thus, researchers are exploring faster-charging, more durable, and more efficient battery materials, and this exploration has led them to nanomaterials. LIBs are critical in modern energy storage, and the use of nanomaterials considerably improves their electrochemical performance. The nanomaterials are useful in the anodes and cathodes of LIBs due to their nanoscale dimensions, which significantly enhance the electrochemical performance. Nanomaterials improve specific capacity, charging capacity, cycle stability, energy density, and efficiency. Some nanomaterials, like graphene and carbon nanotubes, improve ionic conductivity. However, nanostructured materials like silicon nanomaterials can accommodate more lithium ions, better mechanical stability, less volume expansion, and longer cycle life. Thus, adding nanomaterials to LIBs greatly enhances their electrochemical performance, durability, and Coulombic efficiency, opening the door for more sophisticated uses in energy storage and electric vehicles. Further, low ionic conductivity, limited thermal stability, and safety issues result in short-circuiting and thermal runaway, which are common problems with conventional liquid electrolytes in LIBs. These limitations could significantly reduce battery performance and safety. To overcome these challenges, adding nanomaterials to the electrolytes has shown promise as a way to improve mechanical strength and ionic conductivity [21]. Zhao [22] and Poorshakoor et al. [21] studied the advancements in LIBs and demonstrated that nanomaterials enhance battery performance by improving kinetics and stability in anodes (e.g., carbon and silicon), increasing rate capability in cathodes (layered oxides), and boosting conductivity and strength in electrolytes and separators, ultimately optimizing lithium-ion battery components for better efficiency. Nanomaterials enhance battery performance by increasing the electrode’s surface area and reducing ion diffusion, leading to more energy density and electrochemical reaction, as highlighted in various studies on innovative nanostructures and functional materials for battery applications [23]. However, nanomaterials can significantly increase the energy density of LIBs by enhancing ionic conductivity and reducing particle size [3]. Graphene improves energy density and cycling stability due to its larger specific surface area and electrochemical characteristics [4,24]. Silicon nanomaterials have good lithiation properties, improving ion storage and release, which are critical for battery efficiency [25]. According to the thermal stability of the battery, nanomaterials can reduce the risk of overheating associated with LIBs, thereby improving the safety. Nanomaterial can also help in recycling and cleanup of battery components, minimizing environmental impact [26]. While the advancements in nanomaterials present promising solutions for enhancing LIBs, challenges such as scalability and environmental implications of nanomaterial production remain critical considerations for future research and development.

### 4.1. Nanomaterials in the Anode of Lithium-Ion Batteries (LIBs)

The anode is typically made from graphite in traditional Lithium-Ion Batteries (LIBs). However, graphite anodes have a relatively low energy capacity and can suffer from degradation over multiple charge–discharge cycles. Two-dimensional materials based on metal oxides and carbon are the most desirable substances for efficient and long-term energy storage. Advanced carbon nanomaterials improve LIB anodes by increasing energy density, extending their lifespan, and decreasing resistance to dendritic lithium deposition. These materials provide significant improvements over standard graphite, solving critical issues in lithium battery performance. However, silicon nanoparticles, graphene, and CNTs can also be considered to improve anode performance. The subsequent section discusses the above nanoparticles as anodes of LIBs.

#### 4.1.1. Silicon Nanoparticles

Silicon nanoparticles offer higher theoretical specific capacity (~4200 mAhg^−1^) and least potential (~0.4 V) [24] in comparison to graphite. However, they tend to expand and contract during charging and discharging, leading to the mechanical degradation of electrodes and formation of solid electrolyte interphase. The higher volume expansion (~300%) of silicon-based anodes hinders their commercial applications [25]. By using nano-sized silicon or silicon–carbon composites, the volume expansion can be better managed, improving the cycling stability and life. Kim et al. [26] evaluated the efficacy of graphite-based silicon nanoparticles as anodes in LIBs. They focused on composite anodes and blending anodes. They found that the aforesaid anodes exhibited high energy density. Guo et al. [27] selected Si-CNTs as anodes for evaluating the electrochemical performance of LIBs. They found that the electrochemical performance has been improved due to chemical bonding of Si-C. They found excellent capacity retention of 83.3% after 200 cycles. Gim et al. [28] studied a synergetic effect of double-coated silicon in LIBs as an anode material. They evaluated the effect of double-layer coatings on electrochemical performances for different anodes. The authors claimed that the tested silicon nanoparticles enhanced the electrochemical performance of LIB by improving ionic conductivity, cycling stability, and rate capacity. The highest specific capacity (~2300 mAhg^−1^) was found for Si-C-TiO_2_ anodes, at 0.2 C. However, the aforesaid anode retained its outstanding electrochemical performance (specific capacity = 2000 mAhg^−1^) at 0.2 C and 100 cycles. Moreover, they reveal the outstanding capacity retention approximately equal to 82% and better power capability for 10 C. The synergy between the carbon coating and the TiO_2_ layer accounts for this phenomenon. The electrical conductivity increases due to the carbon coating. Similarly, the mechanical robustness and chemical stability are enhanced by the TiO_2_ layer.

**Figure 4:**
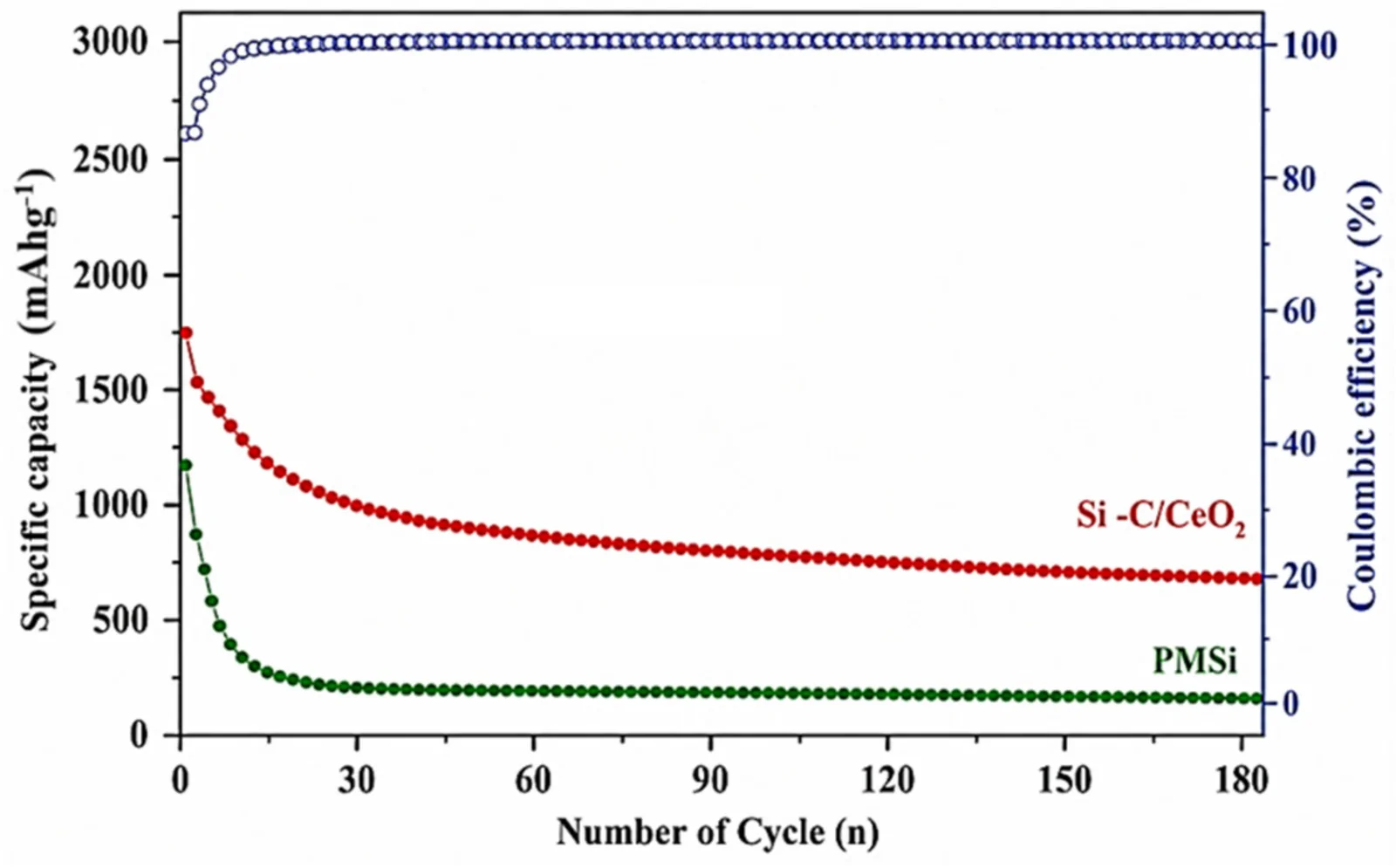
The variation in specific capacity and Coulomb efficiency of Si-C/CeO_2_ composite with cycle number. Data from Ref. [29].

The effect of cerium on silicon–carbon composites (Si-C/CeO_2_) for evaluating the performance of anodes in LIBs was studied by Wang et al. [29]. In their study, the performance of anodes was significantly enhanced due to the addition of Ce to the carbon layers. The underlying reasons are enhancement in the rate capability, ionic conductivity, first Coulombic efficiency, structural stability, and the least development of solid electrolyte interphase (SEI). They found the superior value of specific capacity of 1848.6 mAhg^−1^ for tested composites, and it was maintained at a value of specific capacity of 502.2 mAhg^−1^ beyond 180 cycles, as shown in Figure 4.

Li et al. [30] used CO_2_ in a dealloying process to fabricate silicon–carbon-composite anodes for LIBs. As an anode, the aforementioned composite exhibited excellent electrochemical performance. Additionally, the composite material demonstrated a high capacity rate and specific capacity (714.6 mAhg^−1^). However, there is a limitation of excessive volume changes (300~400%) during the processes of the lithiation and delithiation while using silicon nanomaterials. This makes it difficult to use them practically due to their low Coulombic efficiency (CE) and quick capacity decay. Khazaeli et al. [31] explored the use of holey graphene-silicon nanocomposites as an anode to assess the electrochemical performance of LIBs. To achieve optimal results, they prepared the nanocomposite samples with a silicon-to-holey-graphene-mass ratio of 1:1. Their research indicated a remarkable specific capacity (~785 mAhg^−1^), and an excellent rate of retention (~61%) beyond the cycles of three hundred. Furthermore, the nanocomposite exhibits a specific discharge capacity of 1071 mAhg^−1^ at an elevated current density of 2000 mAhg^−1^. This value of current density is approximately eleven times more in comparison to silicon. Choi et al. [32] developed an innovative anode material by applying a dual coating of TiO_2_ and nitrogen-doped carbon, aimed at enhancing the electrochemical performance of silicon oxide. Their findings reveal an impressive specific capacity (~1427 mAhg^−1^) and rate of retention (~91.4%) for tested conditions of 0.5 C and 100 cycles. The key factors contributing to this performance are the stability provided by TiO_2_ and the improved conductivity and rate capability offered by nitrogen-doped carbon. Table 2 presents an outlook of silicon nanoparticles as an anode in LIBs.

#### 4.1.2. Graphene and Carbon Nanotubes

Graphene is a game-changer for LIBs, especially when used as an anode. Its benefits include enhanced electrochemical performance, greater lithium storage capacity, improved conductivity, and minimized volume expansion. The CNTs have been utilized across a range of applications. Their remarkable electrical conductivity can significantly enhance the rapid charging and discharging capabilities of LIBs, making them ideal candidates for anode materials. They also provide mechanical stability and prevent the anode from breaking down during expansion and contraction. 1D, 2D, and 3D carbon nanostructures provide high electrical conductivity and mechanical stability, thereby improving the transportation of ion [40]. A graphene-based nanocomposite was used as an anode in LIBs by Khan et al. [41]. Their research concluded that the tested nanocomposites could be a better option as an anode in LIBs. The incorporation of graphene also improves the electrochemical performance of LIBs. They have also reported that graphene-based nanocomposites show that an excellent cycle stability. Nguyen et al. [42] focused on composites anodes in LIBs. For this purpose, they dispersed CNT and Si, and reduced GO in an aqueous solution. They have seen that the chemically reduced aforesaid composites improve mechanical resilience. However, the authors have also found a superior electrochemical performance thereby increasing long term cycle performance. The electrochemical performance of titanium niobium oxide (TiNb2O7) was investigated by Aghamohammadi et al. [43]. They utilized graphene and CNT nanomaterials. They synthesized hybrid nanocomposite TiNb2O7–graphene–CNTs in their studies. Their results revealed that the capacity rate and cycle stability of TiNb2O7 were improved. They mentioned the optimum mass ratio of graphene and CNTs (i.e.,1 wt.% graphene and 2 wt.% CNTs), which exhibited a significant cyclic stability of (~161 mAhg^−1^) and current density of (~387 mAg^−1^) at 200 cycles. Zhou et al. [44] prepared a composite (CoS_2_ + rGO + CNT) as anode in LIBs. They utilized the cobalt sulfide (CoS_2_), reduced graphene oxide (rGO), and CNT to make the composite in their research. They found fast ion transport in CoS_2_ + rGO + CNT composites due to strong bonding of CNTs with CoS_2_-rGO. The researchers quoted that the use of CoS_2_ + rGO + CNT composite as anode can increase the life cycle of LIBs. Lu et al. [45] studied graphene-based double-layer carbon-encapsulated silicon as anode in LIB. Their findings revealed an excellent electrochemical performance for LIBs, along with considerable cycle stability and lithium-ion storage. However, they found a significant specific capacity of (~1528.1 mAhg^−1^) and capacity retention of (~45.5%) for 0.1 Ag^−1^. The tested anode could be an alternative method for developing silicon-based anodes.

#### 4.1.3. Transition-Metal Oxide

**Figure 5:**
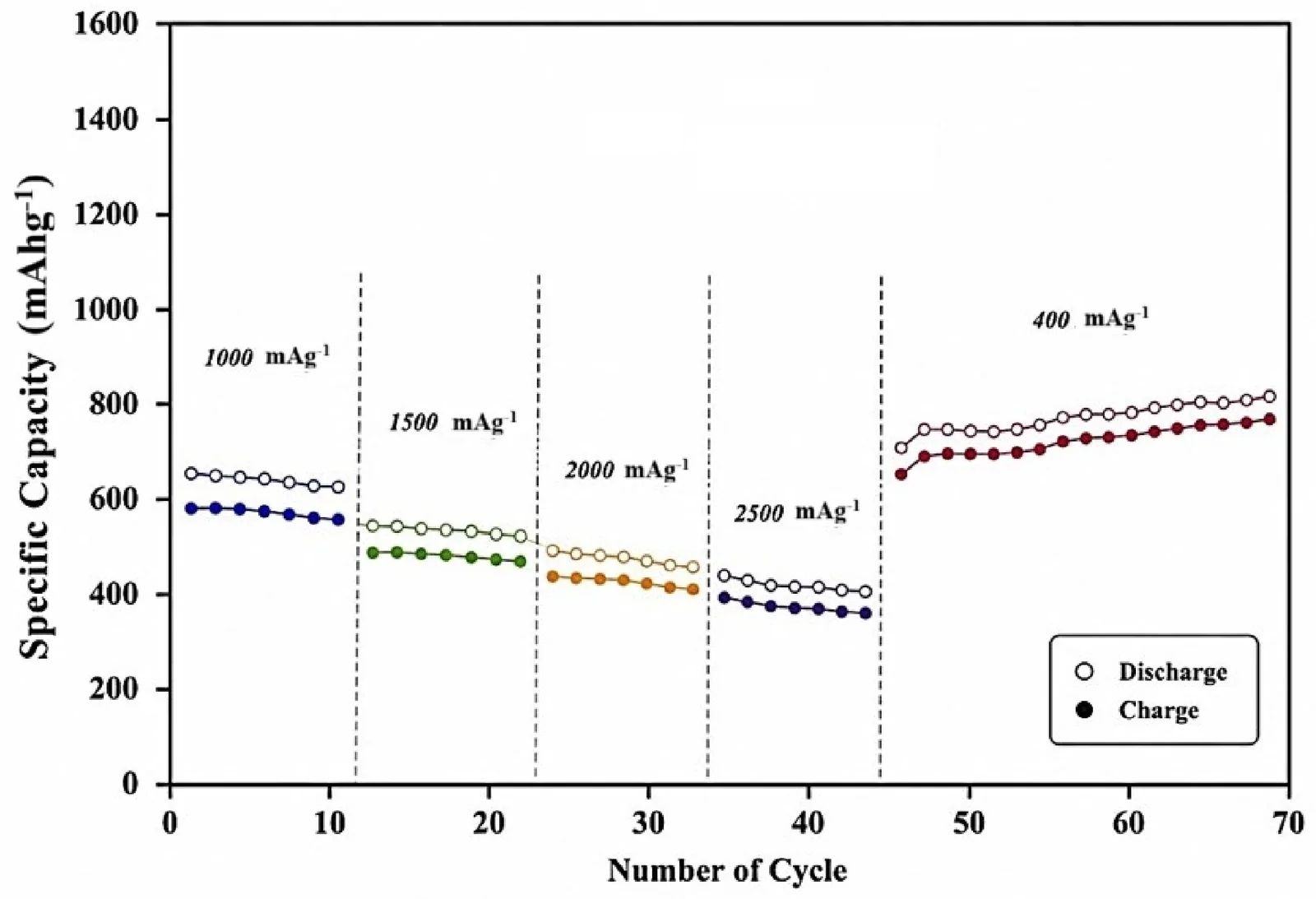
Cycle performance rate of hierarchical porous ZMO microspheres. Data from Ref. [47].

Transition-metal oxides (TMOs) show a significant ability as an anode material for rechargeable batteries (LIBs). They offer a substantial theoretical capacity, affordability, and widespread availability. Materials like titanium trisulfide (TiS_3_) and zinc–manganese Oxide (ZnMn_2_O_4_) improve the specific capacity and cycling stability, addressing the limitations of traditional anode [4]. Nanomaterials in the anode of LIBs include nanostructured silicon, tin oxide composites, and lithium titanium oxide. These materials enhance capacity retention, mitigate volume expansions, and improve charge–discharge performance, addressing challenges like low conductivity and structural stability during cycling [46]. Rong et al. [47] studied hierarchical porous zinc–manganese oxide (ZMO) microspheres in LIBs as anodes. The hierarchical porous ZMO (ZnMn_2_O_4_) microspheres demonstrate significantly superior electrochemical performance compared to both ZnMn2O4 and other transition-metal oxides (TMOs). This happens due to their unique porosity of the structure, which facilitates increased storage of lithium-ion and reduces volume expansion during charging and discharging of LIBs. However, there is notable improvement in cyclic stability. The ZMO has a significant reversible capability of (~723.7 mAhg^−1^) for 400 mA g^−1^ current density. Further, the ZMO demonstrated an outstanding performance rate as shown in Figure 5. Their results reveal specific capacities of 611.7, 515.6, and 463.8 mAhg^−1^ for the range of 1000–2000 mAg^−1^ current densities, as seen in Figure 5.

Liang et al. [48] have employed ZMO (ZnMn_2_O_4_) as anode in LIBs. In their research, they used ZnMn_2_O_4_ sphere/jute as a composite material. Their results show an improved specific capacity (~1501.6 mAhg^−1^) and current density of (~156.8 mAg^−1^) for two hundred cycles. The composite material showcased a remarkable cyclic stability without any loss in capacity. This impressive stability is just because of the unique structure formed during the preparation process, where ZnMn2O4 spheres were integrated with jute biomass porous carbon in the anode material. Fan et al. [49] employed the ZMO microspheres for anodes in LIBs. They reported that ZMO microspheres show an excellent discharging (~1044 mAhg^−1^) for tested conditions. Similarly, the rate capability was found to be 859 mAhg^−1^ at a specific current of 2000 mAg^−1^. This happens due to the porous structure, which enables rapid ionic movement and allows for significant volume expansion and contraction, resulting in outstanding electrochemical performance. An electrochemical performance of LIBs using spinal ZnMn_2_O_4_ nanoparticles was performed by Wang et al. [50]. They claimed an appreciable specific capacity (~500 mAhg^−1^) at a tested current density (~500 mAg^−1^) and 150 cycles. The authors also reported an excellent electrochemical behavior, with novel cycle life. The impressive performance of ZnMn_2_O_4_ can be attributed to its distinctive structure, which features nanoparticle-assembled clusters that create a hollow interior. This structure facilitates rapid electrolyte diffusion and helps to manage volume changes effectively. Moreover, incorporating nanoparticles significantly enhances the electrochemical properties, making ZnMn_2_O_4_ an excellent choice for anodes. Zhang et al. [51] employed ZnMn_2_O_4_ hollow folded microspheres as anodes materials in LIBs. Their findings revealed the considerable specific capacity (~60 mAhg^−1^) for a desired current density of 2 Ag^−1^. They also observed an outstanding life cycle performance of 724 mAhg^−1^ after 400 cycles. This happens due to special structures of the materials. Liu et al. [52] fabricated a pomegranate-structured ZMO nanocomposite, with optimum particle size, as anode for improving the storage capacity of lithium in LIBs. They achieved an outstanding cycle stability of 811 mAhg^−1^ for tested conditions of current density (~0.2 Ag^−1^) and one hundred cycles. Porous flower-like α-Fe_2_O_3_ achieved an impressive initial discharge capacity of 1183 mAhg^−1^ at a rate of 50 mAg^−1^ [53]. Its unique porous structure not only supported excellent cycling stability but also enhanced high-rate performance, highlighting its potential as an affordable transition-metal oxide anode for LIBs.

The binary transition-metal oxide (BTMO) consists of two different transition metals (nickel, cobalt, iron, manganese, and vanadium) combined with oxygen. BTMOs effectively harness the different redox states of two metal ions, resulting in better specific capacitance, energy density, and cycling stability compared to single-metal oxides [54]. Their robust structural integrity, enhanced ionic conductivity, and more redox sites facilitate quicker ion transport, thereby increasing the faster charging/discharging operations in super capacitors [55]. Binary metal oxides (BMOs) consist of two distinct metal elements, typically combining a bivalent alkaline earth metal with a transition metal like molybdate, all within an oxide framework. A detailed comparison of performance of BTMO vs. BMO for LIBs is presented in the Table 3.

#### 4.1.4. Titanium Carbide

A transition-metal carbide like titanium carbide (TiC) is utilized as an anode by the researchers in LIBS. The reasons for this are its lower density, superior conductivity, excellent mechanical properties, and better structural stability [62,63]. Ren and Zhang [64] have chosen TiC-40 as anode in LIBs. Their findings reveal that excellent cycling performance along with Coulombic efficiency as 99.3% at 160 cycles. Huang et al. [63] have prepared a core–shell nanoparticles (TiC–NiO) for anode in LIBs. The aforesaid anode material shows an excellent cycling ability (~507.5 mAhg^−1^) for specific current (~200 mAg^−1^) and 60 cycles. Cao et al. [65] have fabricated a core–shell nanostructured (TiC@C-TiO_2_) for an anode in LIBs. The aforesaid anode exhibited an excellent capacity of lithium storage (~352.8 mAhg^−1^) for a given value (100 mAg^−1^) of specific current. Further, the rate capability was found as 253.6 mAhg^−1^ and 158.1 mAh g^−1^ at 1 Ag^−1^, and 10 Ag^−1^ respectively. However, the core–shell nanostructured anode exhibited an outstanding cycle stability (~150 mAhg^−1^) for 400 cycles.

#### 4.1.5. Nanocomposites

**Figure 6:**
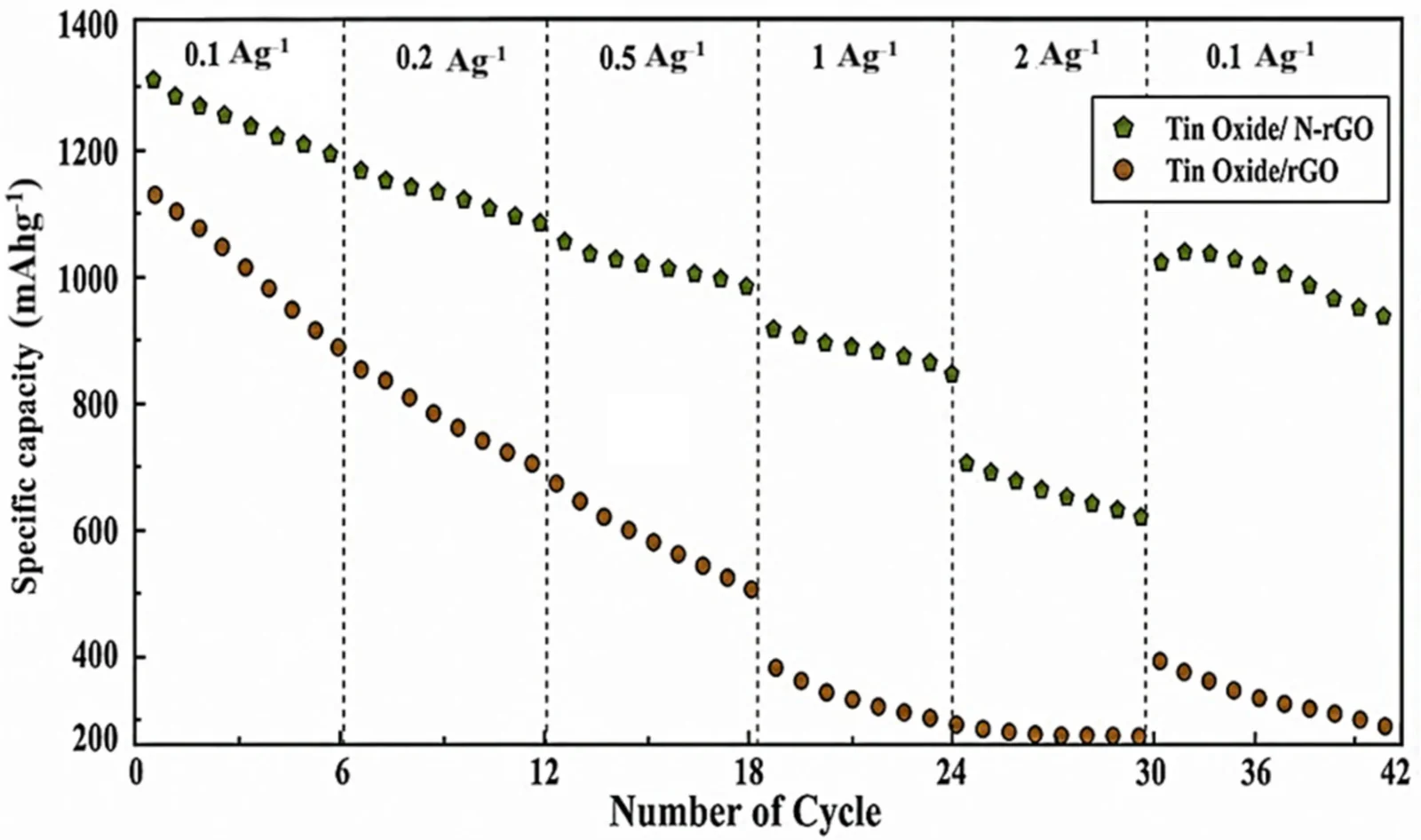
Variation in specific capacity with cycle number. Data from Ref. [68].

The carbon nanocomposite materials are emerging as a promising direction for LIB anodes. These materials are addressing limitations in specific capacity and increased demand of electronic products and EVs [66]. Blending carbon with metal oxides or sulfides creates composites that improve overall performance by combining the strengths of each material [2,40]. Kim et al. [26] utilized blended silicon–graphite (SiG) and its composite for anode in LIBs. Their results show that nanocomposite anode exhibited higher specific capacity (~162 mAhg^−1^) and capacity retention (~73.58%) as compared to blended anode. They have also suggested that a nanocomposite anode could be a better material for achieving the energy for commercial usage. A composite (Si-CNTs) was prepared for anode in LIBs by Guo et al. [27]. They have studied electrochemical performances of Si-CNTs composite and found it relevant due to Si-C bonding. Their results show a significant reversible capacity (~1100.2 mAhg^−1^) at 0.2 Ag^−1^. However, the capacity (~922.4 mAhg^−1^) remains unchanged having capacity retention of (~83.3%) at 200 cycles. Li et al. [24] have developed a composite anode utilizing silicon–graphite nanosheets combined with carbon. Their findings demonstrate a CE value of 91.2% and a rate of capacity retention of 87.5%. Li et al. [67] utilizes a composite of zinc–manganese oxide and porous carbon framework (ZMO-PCF) as anode material in LIBs. They have found an appreciable capacity (reversible) of 760 mAhg^−1^ for the current value of 100 mAg^−1^. At the same time, they have also reported a better rate capability (~500 mAhg^−1^) for a given current density of 800 mAg^−1^. Further, the authors have also reported a favorable cycle stability. It is seen that superior cycling performance of ZMO-PCF at a particular specific current (~1 Ag^−1^). Xu et al. [68] prepared a mesoporous of Tin oxide/N-rGO and Tin oxide/rGO nanocomposites for anode in LIB. They have studied the cycle performance and Coulombic efficiency of the aforesaid nanocomposite. They have reported a higher discharge specific capacity for Tin oxide/N-rGO than Tin oxide/rGO nanocomposites. However, they have compared the electrochemical performance of tin oxide/N-rGO with tin oxide/rGO nanocomposites for the range of specific currents (0.1~2 Ag^−1^), as depicted in Figure 6.

**Figure 7:**
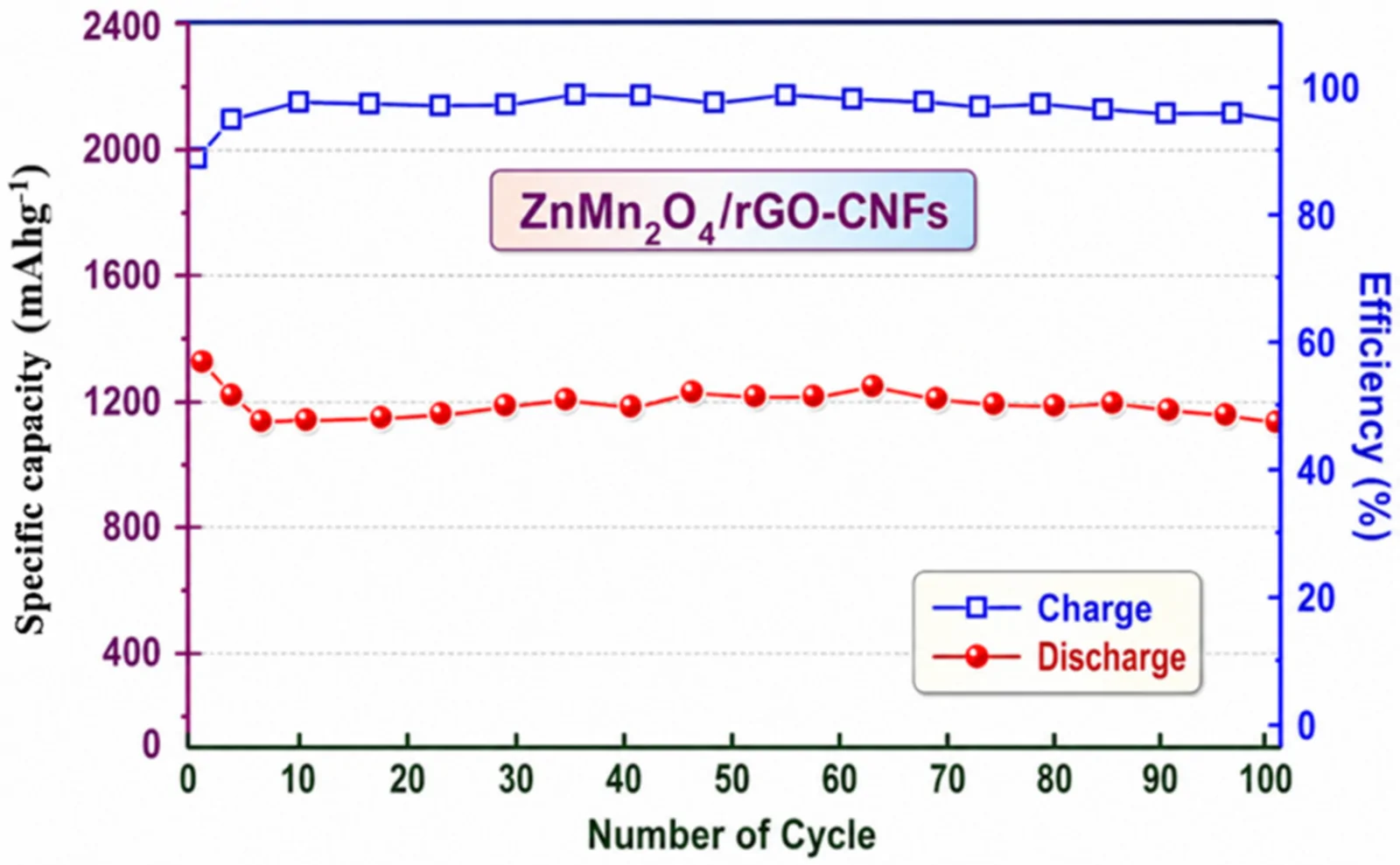
Variation in cycle performance at current density 100 Ag^−1^ of 16 wt.% ZnMn_2_O_4_@rGO-CNFs in LIB. Data from Ref. [71].

Wu et al. [69] synthesized Sb/SnO_2_@C hybrid nanocomposites as anode in LIBs. The hybrid nanocomposite showed remarkable cycle stability. In their study, they have reported a reversible capacity (~302.6 mAhg^−1^) for current (~100 mAg^−1^) and number of cycles (~100). ZnMn_2_O_4_/ZnMnO_3_/ZnO composite was used as anode in LIBs by Liu et al. [70]. They have quoted the maximum rate of capacity of 857.5 mAhg^−1^ for 5 C current and 320 cycles. Gao et al. [71] synthesized the reduced type of graphene oxide (rGO), carbon nanofibers (CNFs) and zinc–manganese oxide (ZnMn_2_O_4_). They formed a new nanocomposite (ZnMn_2_O_4_@rGO-CNFs) and used it as an anode in LIBs. The researchers took 16 wt. % as an optimal sample of the aforesaid nanocomposite. For the optimal sample, the authors designated an initial rate of charge and discharge of 1343 mAhg^−1^ and 2214 mAhg^−1^, respectively, for 100 mAg^−1^ specific current with 60.6% Coulombic efficiency, as presented in Figure 7. The highest Coulombic efficiency was found as 93.4% after ten cycles with excellent electrode reversibility.

### 4.2. Nanomaterials in the Cathode of Lithium-Ion Battery

Nanomaterials in cathodes enhance the performance of LIBs. The reasons behind using nanomaterials in cathodes are high surface area, improved ion transport, faster charging and discharging, enhanced power density, longer life cycle, and improved safety. This is suitable for portable devices and EVs. Nanomaterials have potential to improve energy storage capacity and cycle stability due to their unique properties. Traditionally, lithium cobalt oxide (LiCoO_2_) is used in the cathode, but it has limitations regarding safety and resource availability. Nanomaterials like lithium iron phosphate (LiFePO_4_) exhibit high operating voltage and better thermal stability, leading to more efficient and safer LIBs [72]. Also, nanomaterials like TiS_3_ nanoflakes and ZnMn_2_O_4_ nanobundles enhance the cycle stability and specific capacity of LIBs [4]. Thus, in the subsequent section, various nanomaterials are discussed as cathodes in LIBs.

#### 4.2.1. Nanostructured Lithium Iron Phosphate

The cathodes composed of a lithium iron phosphate (LiFePO_4_) has emerged as a prominent cathode material for LIBs due to its outstanding thermal stability, safety, and cost-effectiveness [73]. Lithium iron phosphate (LFP) improves ionic conductivity and enhances the battery’s performance at a higher rate of charging and discharging for contributing high-power to applications like electric vehicles. LFP exhibited a high capacity rate, cycle stability, thermal stability, and a non-toxicity nature. Kumar et al. [74] developed an ecofriendly LFP for cathodes in LIBs. Their findings revealed high capacity retention (~90%) after 300 cycles. The initial discharge capacity was found to be 130.9 mAhg^−1^. However, LFP is favored for LIBs due to fast charging, thermal stability, safety, and durability. LFP is the most stable material for cathodes at high temperatures in comparison to other materials [75]. Monteiro et al. [76] investigated the performance of LiFePO_4_ as a cathode for LIBs. They presented thermal shutdown compatibilities of cathodes in their study. Their results showed an improved battery performance (~155 mAhg^−1^), with microsphere percentages of 2.5%. Kumar et al. [77] studied the electrochemical behavior of the cathode using LFP. They investigated four different aqueous solutions: two molarity of sodium hydroxide (NaOH), one molarity of sodium sulphate (Na_2_SO_4_), and one and three molarity of potassium hydroxide (KOH). Their results show that electrochemical performance of LFP was optimum with one molarity of potassium hydroxide aqueous solution. LFP exhibits high power capability and thermal stability, with some limitations, such as low lithium-ion diffusion and ionic conductivity [78]. Naduka et al. [79] prepared a composite of LiFePO_4_ (LFP) and Fe_2_O_3_ nanoparticles for a cathode of LIBs. They have conducted electrochemical performance tests with two samples of 1 wt.% and 3 wt.%. In their work, electrochemical performance is improved with high reversible capacity. Ibrahim et al. [80] added Fe_2_O_3_ to phosphate/vanadium glass and glass ceramics for cathodes in LIBs. Their results show an improvement in cyclability due to the addition of Fe_2_O_3_ in LIBs.

#### 4.2.2. Nickel–Manganese–Cobalt Oxide

**Figure 8:**
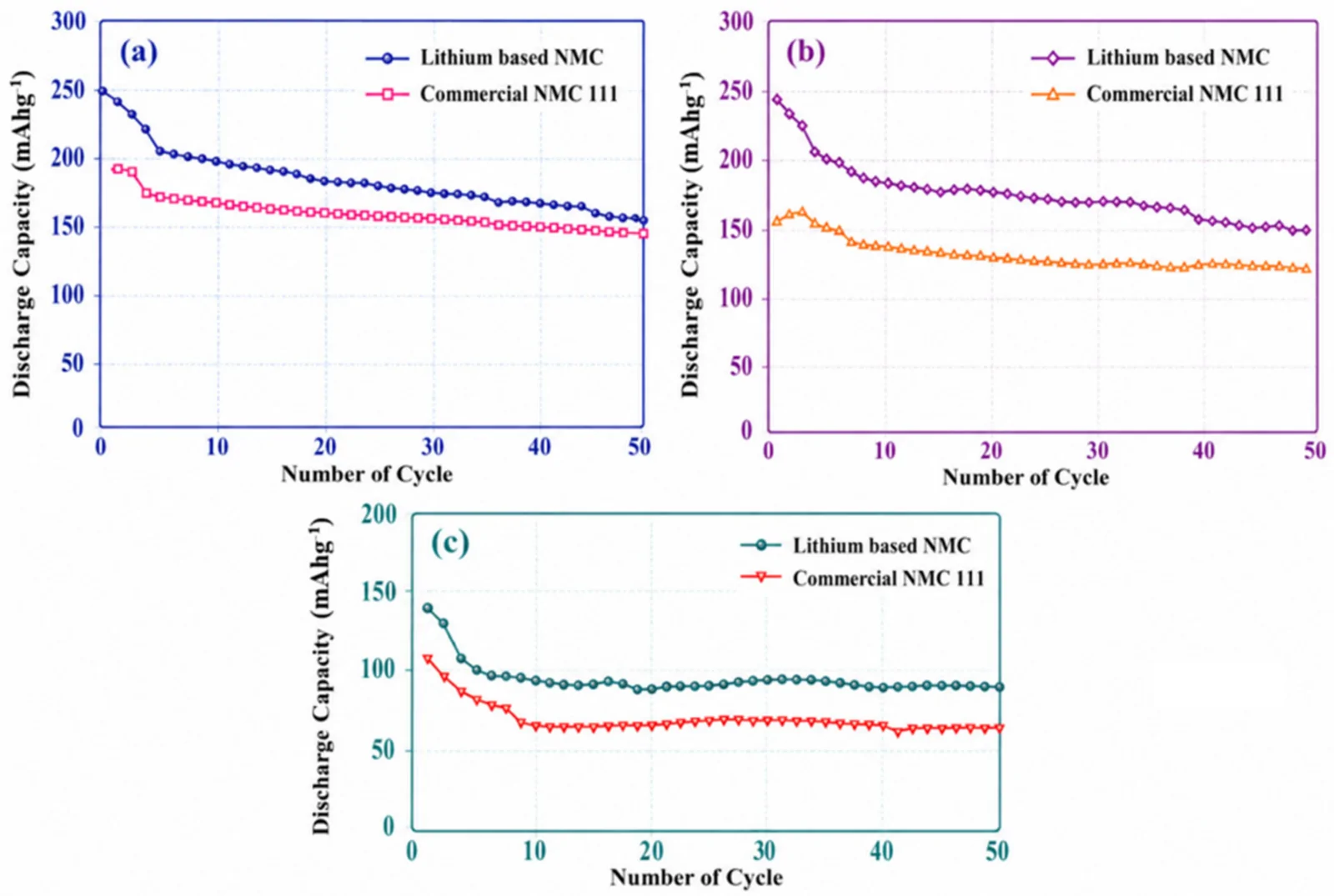
Cycle performance of lithium-based NMC and commercial NMC111 for (**a**) 1C/10 (**b**) 1C/5 and (**c**) 1C sequentially. Data from Ref. [85].

In the new age of batteries technology, nickel–manganese–cobalt oxide (NMC) can be utilized as a material of cathode. NMC is a combination of lithium and transition-metal (TM) oxides. Its chemical formula is in the form of Li (Ni_x_Mn_y_Co_z_)O_2_. NMC exhibited excellent energy density, capacity rate, low cost, eco-friendly, effectiveness, and minimum use of Co [81,82,83]. However, there are some challenges like poor cycle stability and thermal performance associated with NMC. This happens due to increased nickel content. Addition of nanomaterials improves ionic conductivity and stability. Darjazi et al. [84] analyzed the cycle performance of LIBs by using doped NMC materials as cathodes. They reported that doped cathode improves the specific capacity by 232 mAhg^−1^ at 0.1 C. Cetin et al. [85] compared the electrochemical performances of two cathode materials, namely Li-based NMC and NMC111, in coin cells for different conditions. The discharging values of lithium-based NMC were found to be 160, 156, and 96 mAhg^−1^, whereas for NMC111, they were 153 mAhg^−1^, 120 mAhg^−1^, and 72 mAhg^−1^ at 1 C/10, 1 C/5, and 1 C sequentially, as presented in Figure 8a–c.

#### 4.2.3. Transition-Metal Sulfide

Transition-metal sulfide (TMS) is a potential material for cathodes to give them better specific capacity and electrical conductivity in LIBs. Riccardo Rocca et al. [86] used the lithium-rich metal sulfide (Li_2_TiS_3_) as a cathode in LIBs. They reported high energy capacity for the aforesaid cathode material. An effort was made to study the lithium-rich layered titanium sulfides (Li_2_TiS_3_) for cathodes in LIBs by Mespoulie et al. [87]. Their results show an excellent electrochemical performance. The specific energy and reversible capacity were found to be 600 Whkg^−1^ and 265 mAhg^−1^, respectively. Zang et al. [88] used titanium disulfide-coated CNT as a hybrid electrode in LIBs. In their study, they prepared a hybrid electrode made up of TiO_2_ and vertically aligned carbon nanotubes (VANCTs). The researchers concluded that their hybrid electrode showed an excellent capacitance of ~95% after 10,000 cycles.

#### 4.2.4. Zinc–Manganese Oxide

**Figure 9:**
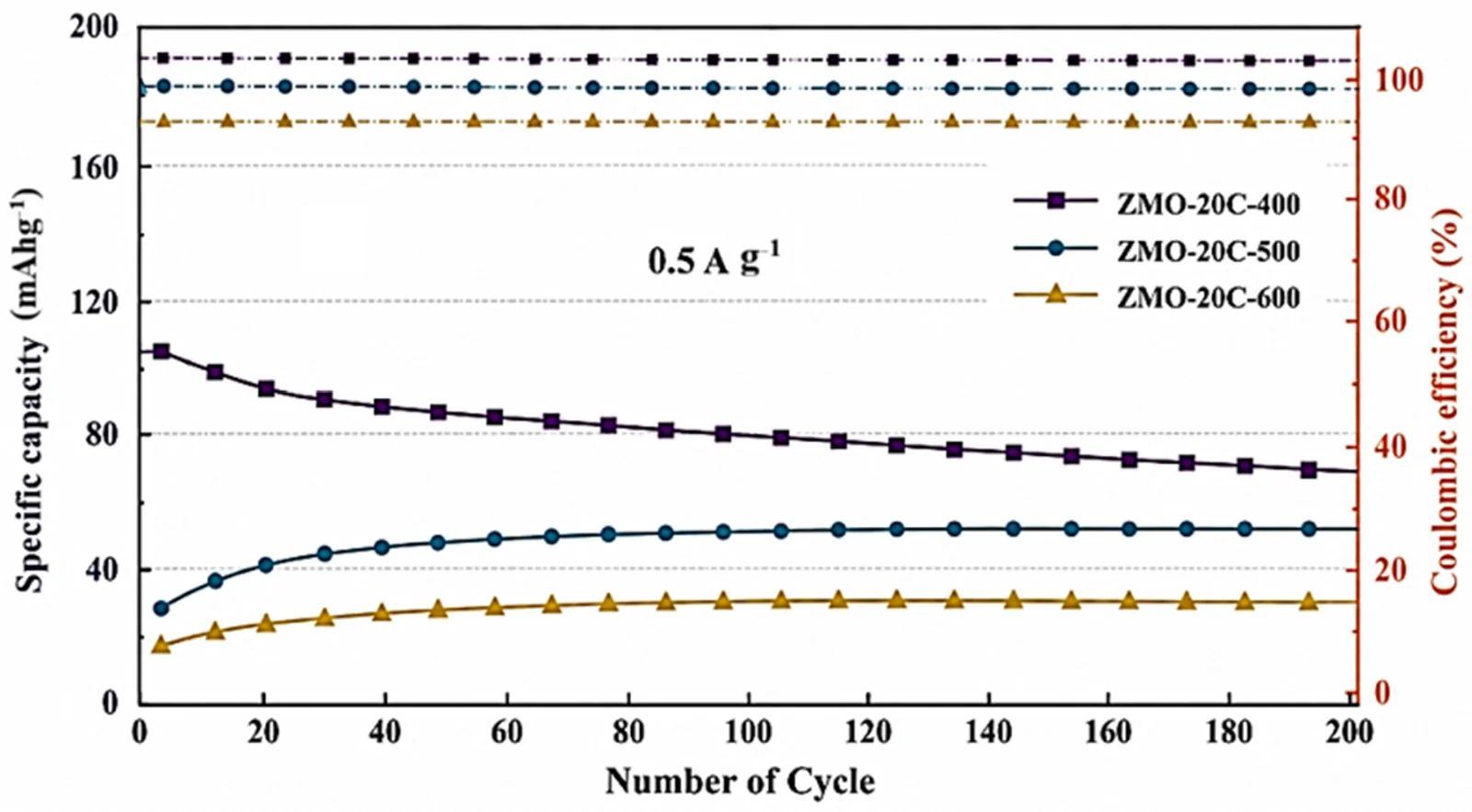
Specific capacity and Coulombic efficiency of ZMO with number of cycles. Data from Ref. [90].

Zinc–manganese oxide (ZMO) improves energy density and safety [4]. A nanocomposite made up of zinc manganite, zinc–manganese oxide (ZnMn_2_O_4_), and reduced graphene oxide (rGO) was synthesized by Kommu [89]. The authors reported that ZMO/rGO exhibits excellent discharge capacity with respect to ZMO. This happens because of enhanced ionic conductivity via the addition of GO. The specific capacity of ZMO/rGO was found to be higher than ZMO. Mei et al. [90] studied the cycling performance of ZMO. According to Figure 9, specific capacity gradually increased due to decrease in calcination temperature. The specific capacity was found to be 87.7 mAhg^−1^, 42.5 mAhg^−1^, and 24.9 mAhg^−1^ for ZMO-20C-400, ZMO-20C-500, and ZMO-20C-600, respectively. The highest specific capacity (125 mAhg^−1^) was presented by ZMO-20C-400 at 0.1 Ag^−1^. Thus, it is concluded that ZMO-20C-400 has the highest storage capacity and Coulombic efficiency.

Table 4 presents experimental findings from the peer-reviewed literature on the benefits of nanomaterials in lithium-ion battery (LIB) cathodes, focusing on capacity, rate performance, and cycling-stability enhancements.

#### 4.2.5. Nanomaterial–MXene Composites

MXenes represent a cutting-edge category of two-dimensional transition-metal carbides and nitrides, first introduced by researchers at Drexel University in 2011 [97]. These materials are chemically denoted as M_n+1_X_n_T_x_, where M represents transition metal (e.g., Ti, Zr, and Hf); X may be nitrogen (N) or carbon (C); and Tx is a functional group, such as –OH, –O, or –F. The variable n ranges from one to four [98,99]. One of the standout features of MXenes is their impressive ionic conductivity and efficient dispersion properties, making them excellent candidates for energy storage applications. However, a challenge they face is their tendency to stack due to Van der Waals forces. This stacking can limit the available active sites and impede ionic conductivity. Nevertheless, there are significant advantages to using MXenes in nanomaterials, including the ability to suppress restacking, facilitate faster Li^+^ diffusion, enhance ionic conductivity, improve specific capacity and rate capability, and increase cycling stability, alongside kinetic reactions. For a more detailed overview, refer to Table 5, which presents a summary of the electrochemical performance of MXene–nanomaterial composites used as cathodes in LIBs.

## 5. Nanomaterials in the Electrolyte

The two main categories of electrolyte materials for LIBs are liquid electrolyte (LE) and solid-state electrolyte (SSE).

### 5.1. Liquid Electrolytes (LEs)

Liquid electrolytes are essential in LIBs. They serve as a medium for transferring lithium-ion (Li^+^) during charging and discharging processes. Liquid electrolytes affect energy density, cycle life, power output, safety, and stability. The conventional liquid electrolyte is usually an organic solvent and lithium salt in a LIB. The liquid electrolytes exhibit several advantages, including outstanding conductivity, stability, less flammability, non-toxicity, and stable SEI formation. However, there are also some challenges, including flammability, dendrite formation, risk of thermal runaway, and SEI breakdown.

Nanoparticles in electrolytes enhance ionic conductivity, rate of capacity, retention, thermal and mechanical stability, and safety (dendrite suppression), contributing to improved performance in LIBs. These achievements overcome the limitations of conventional liquid electrolytes, improving ion transport and overall efficiency, which is important for the overall performance and durability of the battery system [21]. An experimental observation on nanomaterials in conventional electrolytes for LIBs is presented in Table 6.

### 5.2. Solid-State Electrolytes

Solid-state electrolytes (SSEs) are materials which conduct lithium ions in a solid form, such as crystalline, amorphous, or composite. The conventional liquid electrolytes are generally volatile, flammable, and prone to leakage. SSEs are favored over liquid electrolytes because they are non-flammable, do not leak, exhibit reduced dendrite formation, offer higher safety, demonstrate improved electrochemical stability, and provide increased energy density. The addition of nanomaterials improves the salt dissociation and provides faster movement of Li^+^ ions, a stable interface, and uniform lithium deposition, suppressing dendrites with enhanced electrochemical performances (higher capacity, rate capability, and long-term stability). The common nanomaterials used in SSEs are silicon oxide (SiO_2_), aluminum oxide (Al_2_O_3_), titanium oxide (TiO_2_), lithium lanthanum zirconate (LLZO), graphene oxide (GO), reduced graphene oxide (rGO), carbon nanotubes (CNTs), poly (ethylene glycol)-grafted graphene oxide (PEGO), lithium aluminum titanium phosphate (LATP), etc. An experimental observation on different nanoparticles in solid electrolytes for LIBs is given in Table 7.

However, the incorporation of nanoparticles enhances SSEs primarily by reducing polymer crystallinity, facilitating interfacial lithium-ion transport pathways, boosting mechanical strength, stabilizing the lithium/electrolyte interfaces, and preventing dendrite formation. Song et al. [128] integrated 14 wt.% of Li_1.3_Al_0.3_Ti_1.7_(PO_4_)_3_ (LATP) nanoparticles into a poly(ionic liquid)-based composite electrolyte. This incorporation facilitated a more uniform distribution of Li^+^ ions and increased ion transport, while also enhancing the mechanical stability of the electrolyte. The refined electrolyte displayed an electrochemical stability window of 4.9 V and maintained stable Li plating or stripping for over 2000 h at 50 °C. The resulting Li-metal battery achieved a capacity of 145 mAh g^−1^, with a commendable 95% capacity retention after 100 cycles at the same temperature, showcasing significant improvements in both safety and cycling stability. Duong et al. [129] successfully blended 1–3 wt.% commercial TiO_2_ nanoparticles into a PEO-based solid polymer electrolyte through a straightforward and scalable mixing technique. The optimized 1 wt.% TiO_2_ electrolyte demonstrated an impressive ionic conductivity of 3.94 × 10^−3^ Scm^−1^ at 60 °C, along with a Li^+^ transference number of 0.59. Furthermore, the electrolyte facilitated stable lithium plating or stripping for 450 h and achieved a discharge capacity of 126 mAhg^−1^ at 1 C, with a remarkable Coulombic efficiency of 99% after 300 cycles. Saran [130] developed a composite solid electrolyte made from polyethylene oxide (PEO) and tetragonal In-doped lithium lanthanum zirconium oxide (LLZO). This composite solid electrolyte demonstrates an impressive Li^+^ ion transference number of 0.34 and shows good cycling performance at a current density of 0.68 mA cm^−2^ at 40 °C. Furthermore, it achieves an outstanding Li^+^ ion conductivity of 2.40 × 10^−5^ Scm^−1^ at room temperature. The enhanced performance can be attributed to the In-doped LLZO, which facilitates a cubic phase transition that boosts Li^+^ mobility and transport. Based on the author’s research, it appears that there has been limited exploration in the area of solid electrolytes utilizing nanomaterials in the LIBs. Further, a comparison between LEs and SSEs is tabulated in Table 8.

## 6. Current Challenges and Research Gaps

Nanomaterials have demonstrated great potential in enhancing the electrochemical performance of the LIBs, but several challenges still persist:

- High surface reactivity: A larger surface area encourages the development of the solid electrolyte interphase (SEI) and can lead to unwanted reactions with the electrolyte, resulting in a permanent loss of lithium ions (Li^+^).
- Structural instability: Nanostructures can experience coarsening, changes in their shape, or degradation when subjected to repeat cycling.
- Complex synthesis: Achieving precise control at the nanoscale frequently involves expensive, multi-stage processes, which are difficult.
- Manufacturing cost: The production of nanomaterials involves the sophisticated methods of characterization, which can increase the manufacturing costs and, thus, hinder its commercialization.
- Agglomeration: Nanomaterials are generally agglomerate after some time of its usage due to their high surface energy and reduced surface area.
- Poor cycle life: Nanostructures such as silicon experience significant volume fluctuations, exceeding 300%, during cycling. This can result in particle fragmentation and the formation of an unstable solid-electrolyte interphase (SEI).
- Environmental and toxicity issues: Nanomaterials face environmental and toxicity issues during recycling or disposal, thus requiring special handling procedures.

Thus, to overcome these challenges, researchers will have to involve themselves in exploring innovative nanomaterials, refining their production processes, and integrating them into batteries while handling issues related to scalability and cost. Additionally, researchers should be focused on enhancing the safety and environmental sustainability of nanoparticles employed in energy storage systems.

Despite significant advancements in Lithium-Ion Batteriesenhanced by nanomaterials, we still lack a comprehensive understanding of how nanomaterial composition, structure, interfacial chemistry, and electrochemical performance interact across different components, like anodes, cathodes, and electrolytes. There are particularly noteworthy gaps regarding the long-term electrochemical behavior and synergistic redox mechanisms of BMO and BTMO anodes, the simultaneous enhancement of capacity and structural stability in nanostructured cathodes, and the way nanofillers influence Li^+^ transport and the stability of the electrode–electrolyte interface. Additionally, comparing reported performances is often challenging due to differing testing conditions, and practical validations that involve high electrode loading, realistic electrolyte content, and full-cell configurations are still quite limited. Thus, future research should prioritize establishing clear structure–property–performance relationships; advancing multifunctional nanomaterials and stable interfaces; implementing standardized evaluation protocols; and developing scalable, cost-effective, and sustainable fabrication methods to bridge the gap between laboratory findings and the next generation of commercially viable LIBs.

## 7. Conclusions

In the present review, nanomaterials show significant promise for improving the electrochemical performance of next-generation Lithium-Ion Batteries (LIBs). They achieve this through various mechanisms, such as increasing the active surface area, shortening the Li^+^/electron diffusion paths, enhancing charge-transfer kinetics, and ensuring greater structural stability. In the realm of anodes, silicon-based nanomaterials stand out due to their high theoretical capacity, while materials like graphene, carbon nanotubes (CNTs), and other carbon-based nanostructures contribute positively to ionic conductivity and structural integrity. Additionally, binary transition-metal oxides (BTMOs) and binary metal oxides (BMOs) offer appealing alternatives with their multiple redox active sites, compositional versatility, and potential for improved electrochemical kinetics. However, their long-term stability and practical use still require further examination.

On the cathode side, nanostructured lithium iron phosphate (LFP) is known for its excellent safety and cycling stability, while nickel–manganese–cobalt (NMC)-based nanomaterials are particularly attractive for high-energy applications. By employing nanostructuring and surface modification techniques, we can further enhance Li^+^ diffusion, maximize the utilization of active materials, and improve rate capability. For electrolytes, the inclusion of nanofillers like SiO_2_, Al_2_O_3_, TiO_2_, lithium lanthanum titanate (LATP), and lithium lanthanum zirconate (LLZO) can significantly increase ionic transport, mechanical strength, thermal stability, and compatibility at the electrode–electrolyte interface, which makes them highly promising for solid state battery systems.

Nonetheless, deploying nanomaterial-enhanced LIBs faces several challenges, including particle agglomeration, complex interfacial reactions, trade-offs between capacity and stability, inconsistent testing conditions, high production costs, limited scalability, and environmental considerations. Therefore, it is crucial for future research to focus on the rational design of multifunctional anode and cathode nanomaterials, systemic exploration of BMO and BTMO systems, optimization of nanoparticle–electrolyte interfaces, practical electrode loading strategies, standardized testing protocols, scalable and sustainable synthesis methods, and full cell validation. This comprehensive approach is vital for transforming the promising laboratory results of nanomaterials into safe, durable, high-energy-density, fast-charging, and commercially viable LIBs for the future.

## Figures and Tables

**Table 1:** A typical benchmarks for next-generation LIBs [17,18,19,20,21].

Specific Capacity	Capacity Retention (500~100 Cycles)	Coulombic Efficiency	Rate Capability	Ionic Conductivity	Safety
800~1200 mAhg^−1^	>80% (anodes) >90% (cathodes)	More than 99% after initial cycles	Higher than 70% at 5–10 C	>1.0 × 10^−3^ S/cm (at 25 °C)	No thermal runway

**Table 2:** Application of silicon nanoparticles as an anode in LIBs.

S. No.	Key Findings	Specific Limitations	Ref.
1.	High electrochemical performance.	The long-term stability and capacity retention are not discussed. Lack of methods for ensuring the scalability. No comparison of electrochemical performance with existing anode materials, like graphite.	[33]
2.	Lower specific capacity and first CE with decrease in particle size. Greater irreversible capacity loss.	Scale-up challenges. No control on the formation of SEI. Long-term cycling stability. Safety aspects.	[34]
3.	Excellent electrochemical performance. Reduced volume expansion due to nanostructured Si particles. Improved life cycle of the electrodes.	No discussion for trading-off SEI mitigation strategies. No analysis for cast estimation and scalability. Lack of experimental data analysis.	[35]
4.	Enhance electrochemical performance. Suppress the effects of volume expansion while combining Si nanoparticle with the graphitic carbon matrix.	Limited studies were conducted in sodium-ion batteries.	[36]
5.	Potential to replace graphite electrodes in LIBs.	No discussion about volume expansion and improvement in conductivity. Challenging to produce silicon anodes.	[37]
6.	Excellent battery capacity. Anode performance improved.	Specific economical and higher-efficiency silicon nanostructure is required.	[38]
7.	High CE (~100.48%) Good rate of capability due to matrix structure. Specific capacity decreased by 37.24%	The long-term cycling stability (>100 cycles) is not reported. This is required for judging the durability and viability of these materials. No comparison with graphite.	[39]

**Table 3:** Comparison of electrochemical performance of BTMO and BMO in LIBs.

Electrode Materials (BTMO/BMO)	Specific Capacity (mAhg^−1^)	Current Density	Cycling Performance	Remarks	Ref.
ZnMn_2_O_4_	634	100 mAhg^−1^	50	High electrochemical performance	[56]
NiMoO_4_	1020	413 mAhg^−1^	500	Longer cycle life, Excellent electrochemical performance	[57]
CoMoO_4_	1106.3	2.0 Ag^−1^	100	Suitable for anode material, High-performance rate	[58]
MnMoO_4_ sub-nanosheets	965	0.18 Ag^−1^	NA	Good durability and high energy density	[59]
CaMoO_4_	2383	0.5 Ag^−1^	NA	Good cycle stability, high capacity retention, and long life span	[60]
BaMoO_4_	410	0.2 Ag^−1^	NA	100% Coulombic efficiency at 0.2 Ag^−1^ current density and high discharge capacity	[61]

NA: not available.

**Table 4:** Summary of experimental findings on nanomaterials in LIBs cathodes.

S. No.	Nanomaterials	Cathode Composition	Voltage Window	Current Density/ C-Rate	Cycle Number	Key Findings	Ref.
1.	Graphene oxide sheets enveloping LiMn_2_O_4_ nanorods	LiMn_2_O_4_/GO	3.0–4.3 V	0.05 C	100	High specific capacity rate (~130 mAhg^−1^ at 0.05 C). Outstanding capacity retention (~87% after 100 cycle). High structural stability. High Coulombic efficiency (~98% after 100 cycles).	[91]
2.	Nitrogen (N)-doped carbon nanomaterials	LiFePO_4_/N-doped carbon	2.5–4.1 V	0.1–60 C	100	Increased ionic conductivity. High C-rate capability Enhanced cycling stability.	[92]
3.	Al_2_O_3_ surface coating	LiCoO_2_/Al_2_O_3_	Not specified	Not specified	180	Improved thermal stability. High capacity retention (~98.6%) after 180 cycles.	[93]
4.	CeO_2_ nanoparticles	LiCoO_2_/CeO_2_	Not specified	Not specified	50	Improved capacity (138.7 mAhg^−1^). High retention (~89%) after 50 cycles.	[93]
5.	Al_2_O_3_-doped ZnO	LiCoO_2_/Al_2_O_3_-ZnO	Not specified	Not specified	150	High capacity rate (~193 mAhg^−1^). High retention (~90%) after 150 cycles.	[93]
6.	Carbon-coated LiFePO_4_ nanowires	LiFePO_4_/CNT	2.5–4.2 V	High rate	NA	High capacity rate (~132.8 mAhg^−1^) at 0.2 C. High performance (at low temperature).	[94]
7.	Core–shell NMC microparticles	Li(Ni_0.8_Co_0.1_Mn_0.1_)O_2_	Not specified	Not specified	NA	High structural stability. Enhanced electrochemical performance.	[95]
8.	AlPO_4_ coating	LiCoO_2_/AlPO_4_	Not specified	Not specified	NA	High rate of capacity (~233 mAhg^−1^). High capacity retention (~64%)	[93]
9.	MOF-derived porous carbon nanofibers	MOF/nanocomposite	Not specified	Not specified	NA	High capacity rate. Improved cycling stability.	[96]

NA: not available.

**Table 5:** Experimental electrochemical performance of nanomaterial–MXene composites for LIB cathodes.

S. No.	Cathode Composition	Specific Capacity	Cycling Life/Capacity Retention	Rate Capability	Observations	Ref.
1.	LFP nanoplates decorated MXene (LFP@C/MXene composite)	156.6 mAhg^−1^ for 500 cycles at 1 C	94.8% for 500 cycles	139 mAhg^−1^ at 20 C	This electrode composition could be useful for faster charging of LIBs.	[100]
2.	LFP/MXene/KB	120 mAhg^−1^ at 10 C	Good stability	120 mAhg^−1^, at 10 C and 133 mAhg^−1^ at −40 °C/0.1 C	The ionic conductivity of LFP is improved by MXene due to its hydrophilicity Ketjen black (KB) was mixed to suppress the restacking of MXene. Beneficial for low-temperature performance.	[101]
3.	LMO/MXene nanocomposite	111 mAhg^−1^ at 0.1 C	95.2% after 100 cycles	Not reported	Nanocomposite based on MXene could be beneficial as cathodes in LIBs with high performance.	[102]
4.	LiNi_0.45_Mn_1.45_Y_0.05_Ti_0.05_O_4_@MXene nanocomposite	128.29 mAhg^−1^	95.12% retention after 500 cycles at 1 C	Improved rate capability	Improvement in Li^+^ ion diffusion. Increased interfacial stability.	[103]
5.	Ti_2_C MXene nanostructures	174.77 mAhg^−1^ discharge capacity at 1 C	90.60% after 100 cycles	Capacity increased by 74.2%	Enhances long term stability and electrochemical performance. Safer cathode material for LIBs	[104]
6.	Li-rich Mn-based composite	Not specified	87.3% after 300 cycles at 5 C	158.3 mAhg^−1^ at 5 C	Reduces electrochemical polarization. Improves rate capability and cycle stability.	[105]

Nanomaterial–MXene composites present a valuable opportunity for enhancing the performance of LIB cathodes. They can significantly improve factors like ion transport, interfacial stability, rate capability, specific capacity, and cycling stability. Nevertheless, addressing the systematic optimization of the nanomaterial–MXene composition, interfacial chemistry, practical electrode loading, and achieving high electrochemical performance is still a major challenge we need to overcome.

**Table 6:** Experimental observation on nanomaterials in conventional electrolytes for lithium-ion batteries.

S. No.	Nanoparticles	Electrolytes	Experimental Findings	Ref.
1.	Copper/lithium titanate oxide (Cu/Li_4_Ti_5_O_12_)	PVA/PVP-Cu/Li_4_Ti_5_O_12_	Improved ionic conductivity. Faster charging and discharging. Enhanced stability.	[106]
2.	Nickel and sulfur	Lithium manganese oxide spinel (LiMn_1.9_Ni_0.1_O_3.99_S_0.01_)	Outstanding Coulombic efficiency. Enhanced rate capability and stability. Good electrochemical performance.	[107]
3.	Boron nitride nanosheet (BNNS)	Nano-colloidal electrolytes	Reduced Li dendrites formation. Outstanding rate performance.	[108]
4.	Boron nitride nanotubes (BNNTs)	1 M LiPF_6_ in ethylene carbonate/dimethyl carbonate	Enhanced conductivity (~30%). Highest Coulombic efficiency (~99.6%). Excellent reversible capacities. Highest cyclic retention.	[109]
5.	Titanium oxide (TiO_2_)	TiO_2_-based films in nitride	High discharge capacity (>1100 μA/hcm^2^) was maintained after 100 cycles.	[110]
6.	Silicon oxide (SiO_2_)	Composite polymer	Highest ionic conductivity (1.68 × 10^−4^ S/cm) at 30 °C.	[111]
7.	Polyhedral oligomeric silsesquioxane functionalized with poly(ethylene glycol) (POSS-PEG)	nanocomposite polymer electrolytes (NCPE)	Ionic conductivity increased by two times (~3.98 × 10^−6^ S/cm). Improved electrochemical performance.	[112]
8.	Silicon oxide (SiO_2_)	NCPE	Highest ionic conductivity (6.2 × 10^−5^ S/cm) for 7.5 wt.%. Excellent thermal stability.	[113]

**Table 7:** Experimental observation on nanoparticles in solid electrolytes for lithium-ion batteries.

S. No.	Nanoparticles	Electrolytes	Experimental Findings	Ref.
1.	GO	PVDF-HFP/PMIA	Enhanced mechanical and thermal stability. High capacity retention (~84.7%) at 0.5 C after 200 cycles.	[114]
2.	GO	LiClO_4_–polyacrylonitrile polymer electrolyte	High Ionic conductivity (~4.03 × 10^−4^ S/cm) at 30 °C.	[115]
3.	SiO_2_ nanospheres	PEO solid polymer electrolyte	Improved ionic conductivity (1.2 × 10^−3^ S/cm, 4.4 × 10^−5^ S/cm at 60 °C and 30 °C, respectively). Extended electrochemical stability (~5.5 V).	[116]
4.	SiO_2_	PEO-LiClO_4_	Increased ionic conductivity of (~1.1 × 10^−4^ S/cm) at 30 °C. High capacity retention (~70%) after 100 cycles. High Coulombic efficiency (~99%).	[117]
5.	In situ synthesized SiO_2_	Composite solid polymer electrolyte (CPE)	Best ionic conductivity (~1.03 × 10^−3^ S/cm) at 60 °C. High electrochemical stability (~5.5 V).	[118]
6.	SiO_2_ and Al_2_O_3_	PEO-based polymer electrolytes	Improved ionic conductivity (10^−7^~10^−5^ S/cm). Improved compatibility and stability.	[119]
7.	Al_2_O_3_	PAALi-based CPEs	Increased ionic conductivity and mechanical properties.	[120]
8.	BaTiO_3_ nanoparticles	PEO-based SPEs	Enhanced ionic conductivity (2.2 × 10^−5^ S/cm and 1.9 × 10^−3^ S/cm at 25 °C and 80 °C, respectively) with 8% wt. High specific capacity of approximately 97.8% at 80 °C after 50 cycles.	[121]
9.	TiO_2_	Nanocomposite polymer gel	Ionic conductivity was enhanced up to 4.8 mS/cm at room temperature.	[122]
10.	Nanosized functional particles	NDCE	Improved battery performance.	[123]
11.	Methacrylic polymeric	Single-ion polymer electrolytes	High ionic conductivity (2.9 × 10^−4^~2.3 × 10^−5^ S/cm). Outstanding electrochemical properties.	[124]
12.	SiO_2_	NCPE	Enhanced ionic conductivity (~6.2 × 10^−5^ S/cm) with 7.5 wt.%. Excellent thermal stability (~334 °C)	[113]
13.	SiO_2_	Polymer electrolytes	Ionic conductivity decreases below their crystallization temperature.	[125]
14.	Li_1.4_Al_0.4_Ti_1.6_(PO_4_)_3_ (LATP)	LATP/LiMn_2_O_4_	Enhanced cycling stability and rate capability. High conductivity.	[126]
15.	SiO_2_	LiBF_4_ in EMIMBF_4_	Improved ionic conductivity (~0.274 mScm^−1^) at 30 °C	[127]

**Table 8:** Comparison between liquid electrolytes and solid-state electrolytes.

S. No.	Aspects	Liquid Electrolytes	Solid-State Electrolytes	References
1.	Safety/thermal stability	Flammable, prone to thermal runaway at ~120 °C	Non-flammable, thermally stable (up to >300 °C)	[131]
2.	Ionic conductivity	High, at room temperature	Low, at room temperature	[132,133]
3.	Energy density	Lower	Higher	[131]
4.	Cycle life	Less	More	[134]
5.	Dendrite suppression	Poor	Good	[131,134]

## Data Availability

No new data were created or analyzed in this study. Data sharing is not applicable to this article.

## Abbreviations

The following abbreviations are used in this manuscript:

BNNS	Boron nitride nanosheet
BNNT	boron nitride nanotubes
CNS	Carbon nanospheres
CNT	Carbon nanotube
CPEs	Composite polymer electrolytes
EV	Electric vehicle
GO	Graphene oxide
HFP	Hexafluoropropylene
LFP	Lithium phosphate
LIP	Lithium iron phosphate
LIB	Lithium-ion battery
LiPON	Lithium phosphorus oxynitride
LLZO	Lithium lanthanum zirconate
LMO	Lithium manganese oxide
LTO	Lithium titanate
MOF	Metal–organic frameworks
NCA	Nickel–cobalt–aluminum oxide
NCM	Nickel–cobalt–manganese
NCPE	Nanocomposite polymer electrolyte
NDCE	Nanoparticle-dispersed colloidal electrolyte
NMC	Nickel–manganese–cobalt oxide
PAALi	Poly(lithium acrylate)
PEGO	Poly(ethylene glycol)-grafted graphene oxide
PEO	Poly(ethylene oxide)
PMIA	Poly-m-phenyleneisophthalamide
LCO	Lithium cobalt oxide
PVDF	Poly vinylidene fluoride
SEI	Solid electrolyte interphase
SPEs	Solid polymer electrolytes
SSB	Solid state battery
ZMO	Zinc–manganese oxide
